# Attenuated sensitivity to the emotions of others by insular lesion

**DOI:** 10.3389/fpsyg.2015.01314

**Published:** 2015-09-01

**Authors:** Yuri Terasawa, Yoshiko Kurosaki, Yukio Ibata, Yoshiya Moriguchi, Satoshi Umeda

**Affiliations:** ^1^Department of Psychology, Keio UniversityTokyo, Japan; ^2^Department of Communication Disorders, Health Sciences University of HokkaidoHokkaido, Japan; ^3^Department of Neurosurgery, Nasu Red Cross HospitalTochigi, Japan; ^4^Department of Psychophysiology, National Institute of Mental Health, National Center of Neurology and PsychiatryTokyo, Japan

**Keywords:** insula, interoceptive accuracy, facial expression, arousal level, heartbeat perception task

## Abstract

The insular cortex has been considered to be the neural base of visceral sensation for many years. Previous studies in psychology and cognitive neuroscience have accumulated evidence indicating that interoception is an essential factor in the subjective feeling of emotion. Recent neuroimaging studies have demonstrated that anterior insular cortex activation is associated with accessing interoceptive information and underpinning the subjective experience of emotional state. Only a small number of studies have focused on the influence of insular damage on emotion processing and interoceptive awareness. Moreover, disparate hypotheses have been proposed for the alteration of emotion processing by insular lesions. Some studies show that insular lesions yield an inability for understanding and representing disgust exclusively, but other studies suggest that such lesions modulate arousal and valence judgments for both positive and negative emotions. In this study, we examined the alteration in emotion recognition in three right insular and adjacent area damaged cases with well-preserved higher cognitive function. Participants performed an experimental task using morphed photos that ranged between neutral and emotional facial expressions (i.e., anger, sadness, disgust, and happiness). Recognition rates of particular emotions were calculated to measure emotional sensitivity. In addition, they performed heartbeat perception task for measuring interoceptive accuracy. The cases identified emotions that have high arousal level (e.g., anger) as less aroused emotions (e.g., sadness) and a case showed remarkably low interoceptive accuracy. The current results show that insular lesions lead to attenuated emotional sensitivity across emotions, rather than category-specific impairments such as to disgust. Despite the small number of cases, our findings suggest that the insular cortex modulates recognition of emotional saliency and mediates interoceptive and emotional awareness.

## Introduction

Our emotional experience is well associated with internal bodily change. We have a racing pulse or sweaty palms when we are excited. The peripheral theory of emotion (James, 1884; Lange, 1885/1992) proposed that “our feeling of the same changes as they occur is the emotion.” Although the theory triggered long discussion, the findings of recent psychological and brain imaging studies have indicated that we refer to our internal bodily state when we are aware of our emotional state. Additionally, our internal state modulates our emotional experience (Bechara et al., 1996; Pollatos et al., 2005; Lane, 2008; Dunn et al., 2010; Terasawa et al., 2013a,b).

The perception of afferent information arising from anywhere and everywhere within the body has been termed “interoception” (Sherrington, 1906; Cameron, 2001). Some methods have been developed as measurements of an individual’s sensitivity to perceive interoception, such as the heartbeat perception task (Schandry, 1981) and water load test (Herbert et al., 2012). Some questionnaires such as the Autonomic Perception Questionnaire (Mandler et al., 1958), the Body Perception Questionnaire (Porges, 1993), and The Multidimensional Assessment of Interoceptive Awareness (Mehling et al., 2012) are known as indices of interoceptive sensibility (Garfinkel et al., 2015). Some studies have suggested that sensitivity to interoception represents the disposition of a person’s emotional experience, such as its intensity (Critchley et al., 2004; Pollatos et al., 2005; Werner et al., 2009) or the tendency to focus on arousal (Barrett et al., 2004). Furthermore, a previous study of ours showed that interoceptive sensitivity predicts sensitivity to the emotions of others (Terasawa et al., 2014). These findings indicate that the way in which interoceptive information is processed can affect emotional experience in daily life.

Neuroimaging studies also support the notion that interoceptive sensitivity is closely connected with emotional experience. Activation of anterior insular cortex is commonly observed in studies investigating emotional experience or empathy (Lamm and Singer, 2010; Lee and Siegle, 2012; Gu et al., 2013). Anterior insular activation is also found when participants are aware of their internal bodily state (Evans et al., 2002; Critchley et al., 2004; Hamaguchi et al., 2004). Our previous study confirmed that both interoceptive and emotional awareness recruited right anterior insular activation, and the activation mediated interoception and disposition of social anxiety (Terasawa et al., 2013b). These findings support the hypothesis that the integration of interoception and the interpretation of environmental information yields the subjective feeling of emotion and that the insular cortex is one of the critical regions housing this mechanism.

If interoception plays a role in the foundation of subjective emotion, what changes can be observed when the insular cortex is damaged? Case NK had a selective left middle insula lesion by cerebral infarction and showed difficulties in recognizing and experiencing disgust, even though memory and intelligence were well preserved (Calder et al., 2000). Several studies support this finding (Adolphs et al., 2003; Borg et al., 2013). The origin of disgust is considered to be a biological signal for preventing the intake of rotten foods (Susskind et al., 2008). Furthermore, Penfield found that people felt sensation in their gastrointestinal organs when electrical stimulation was applied to the insular cortex during a neurosurgical operation (Penfield and Faulk, 1955). Reported abdominal sensation, pain, and nausea by stimulation of the insula suggest that the insula plays a key role in representing interoception. These facts indicate that the insular cortex is important not only in interoceptive processing, but also in generating emotional experience that relates to bodily sensation.

However, the accumulation of neuropsychological studies on emotional processing revealed that not all areas of the insular cortex simply subserve the feeling of disgust. A recent neuropsychological study of 15 insular cortex lesion cases showed that the ability to recognize fear, happiness, and surprise was affected, although there was no change in disgust (Boucher et al., 2015). Adolphs et al. (2000) and Dal Monte et al. (2013) examined the recognition of six basic emotions in over 100 patients with lesions and identified several regions that when damaged led to the decline of emotion recognition performance; these included the medial prefrontal cortex, cingulate cortex, supramarginal gyrus, and insular cortex. Patients with insular lesions showed lower performance on discrimination between negative emotions, rather than a specific deficit for feeling disgust.

Recent studies presented findings those were contrary to expectations from previous studies. For example, Damasio et al. (2012) reported the bilateral insular lesion cases showed normal ability to feel emotion and self-awareness. Furthermore, Couto et al. (2013) found a minimal impact of insular cortical lesions when compared subcortical lesion cases. These studies suggest that the insular cortex is not solely involved in the neural substrates of emotional awareness, but rather that subcortical neural networks between the insula and other regions, such as the thalamus, should be considered. Furthermore, these studies highlight the importance of accurate documentation of the effects of insular lesions on emotional recognition ability beyond the performance of basic emotion category recognition.

A previous study asked patients with insular lesions to evaluate the arousal level of emotion-inducing pictures and observed that the patients reported a lower arousal level for the pictures regardless of valence (Berntson et al., 2011). Because arousal level and the activity of the sympathetic nervous system are closely connected, deviation from the homeostatic state of sympathetic activity and perceiving the deviation can lead to recognition of the arousal level. The circumplex model of emotion posits two dimensions for defining emotions, arousal, and valence (Russell, 1980; Kuppens et al., 2013). Emotions are plotted on the plane defined by arousal and valence. Sadness, anger, and disgust are plotted closely to each other on the valence dimension; however, arousal level is the difference between these emotions. The arousal level for anger is the highest, but for sadness it is the lowest and disgust is in the middle. The insular cortex is considered to be one neural substrate for representing arousal level (Lewis et al., 2007), and decreased blood flow in this region yields blunting of interoception (Khalsa et al., 2009). These findings may support the hypotheses that insular cortical and/or subcortical lesions impair interoception, which underlies the recognition of arousal level, and leads to lower performance for discrimination between negative emotions.

On the basis of this hypothesis, we examined the effects on emotional experience following brain lesions including those in the right anterior insula cortex. We measured sensitivity to other’s emotion using morphed continua photos that ranged between a neutral and an emotional facial expression, and revealed that the sensitivity was predicted by the interoceptive accuracy of individuals (Terasawa et al., 2014). These results may suggest that emotional experience is affected by the perception of internal bodily changes triggered by emotional expression. In the present study, we examined changes in emotional experience in patients with right anterior insular lesions by the experimental task, and discuss its influence on their daily life.

## Materials and Methods

The study was performed with the approval of the Keio University Research Ethics Committee (No. 09006). Before participating in the study, all cases were informed (1) of the purpose and procedure of the study and (2) that they were able to cease their participation in the study at any time. All cases signed a written informed consent form.

### Cases

Three patients with lesions participated the study. They were outpatients of the neurosurgery department of the Nasu Red Cross Hospital.

#### Case A

**FIGURE 1:**
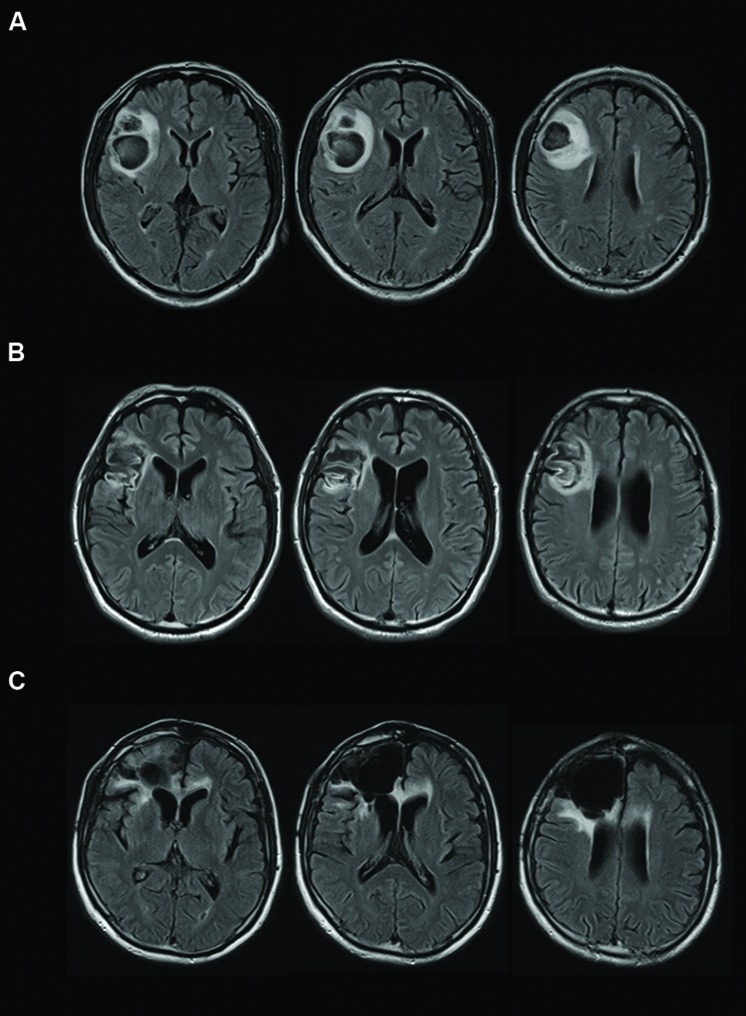
**Transverse magnetic resonance imaging with FLAIR acquisition of the brains of the cases.** Left sides of images correspond to the right side of the brain. **(A)** Case A, **(B)** Case B, and **(C)** Case C.

Case A, a right-handed 61-year-old man, experienced convulsive seizure by cerebral infarction when he was 59 years old. Right dorsolateral prefrontal cortex (DLPFC) and anterior insula cortex were resected by a hematoma evacuation operation (**Figure 1A**). The lesion area included the cortical and subcortical anterior to middle insula and ranged from ventral to dorsal parts. Three-dimensional magnetic resonance angiography showed no occlusions of the major cerebral arteries. Case A has a past history of myocardial infarct, diabetes mellitus, hypertension, paroxysmal atrial fibrillation, and symptomatic epilepsy. Motor function, perception, and language abilities are well preserved. Orientation, memory, working memory, and intelligence are also preserved, but Perseveration error on the Wisconsin card-sorting task (WCST) was observed [Category achieved (CA) : 0, Perseverative errors of the Nelson type (PEN) : 25, Difficulties of maintaining set (DMS) : 0].

Case A reported no change in memory and motor function, but he described himself as having more impatience and being duller compared with his premorbid level. He is still working at a supermarket 5 days a week. According to his family, Case A became easy to anger, disinhibited, and apathetic. Case A showed a poor understanding of others’ intentions or facial expressions. His thermal sensations became dull and he never felt hot. These reports indicated that even though his memory ability and intelligence were preserved, Case A had difficulty in controlling his own emotions.

#### Case B

Case B, a right-handed 45-year-old man, experienced aneurysmal subarachnoid hemorrhage of the middle cerebral artery when he was 44 years old. He underwent surgical clipping. Magnetic resonance imaging showed hemorrhagic contusion of the temporal pole and decreased cerebral perfusion was observed at the right dorsolateral frontal cortex and the anterior insular cortex (**Figure 1B**). The lesion area included the cortical and subcortical anterior to middle insula and ranged from the ventral to dorsal parts. Motor function, perception, and language abilities are well preserved. Orientation, memory, working memory, and intelligence are also preserved, but Perseveration error on the WCST was observed (CA: 2, PEN: 25, DMS: 1). Lower performances of verbal fluency test and delayed memory recall were observed.

Case B reported that he has mellowed and is easily tired. He never feels anxiety about anything, and cannot understand people who feel anxiety at all. According to his family and doctor, Case B shows a poor understanding of others’ facial expressions. Additionally, he has an attitude of indifference toward his own clinical condition. The indifference may coincide with the lower anxiety trait.

#### Case C

Case C, a right-handed 66-year-old man, experienced convulsive seizure and left hemiparesis when he was 60 years old. Brain edema of the right frontal white matter was observed by computed tomography scan. Pathological changes were observed on the surface of the right frontal and premotor areas, and the right frontal lobe including the right anterior insula was resected (**Figure 1C**). The lesion area included the cortical and subcortical anterior insula and ranged from the ventral to dorsal parts. Then, Case C was diagnosed with oligodendroglioma. Orientation, memory, working memory and response inhibition, and sustained attention are preserved, but lower performance on the WCST (CA: 0, PEN: 21, DMS: 0) and verbal fluency task were identified. Motor function, perception, language abilities, and communication abilities are well preserved. He went back to his work.

Case C reported that he frequently experienced action slips and sometimes lost his intention for action. For example, he required much more effort for concentrating on reading books compared with symptom onset. However, he and his family thought this impairment did not significantly impact on his daily life. He had myocardial infarction four times for 4 years since the neurosurgical procedure. However, he traveled through Japan by bicycle on his own and also trained for a marathon, regardless of the heart attack risk. Surprisingly, he reported that he hardly felt anxious or at risk when performing such an exercise.

### Age Matched Healthy Control

Fourteen males (mean age 56.7 ± 3.63 years) participated and completed the heartbeat perception task and the emotional sensitivity task. No participants had any psychiatric disorders. Mini Mental State Examination (MMSE) was conducted and confirmed that their score exceeded 27 points (mean 28.9 ± 0.95), indicating no subjects with cognitive impairment.

### Materials and Procedures

#### Heartbeat Perception Task

Only Cases A and C participated the Heartbeat Perception Task. The task was based on a task developed by Schandry (1981) and Ehlers and Breuer (1992) for examining interoceptive accuracy. The task has been used in many previous studies. A detailed description of the task can be found in our previous study (Terasawa et al., 2014).

Heartbeats were measured using a pulse oximeter (Polymate AP1542, TEAC, Tokyo, Japan) on the fingertips during specific periods of time. Participants were asked to count the number of times that they felt their own heartbeat during the measurement period. Discrepancies between the number of reported and actual heartbeats during the measurement period were calculated based on the formula used to define heartbeat perception error rates by Ehlers and Breuer (1992): (actual heartbeats – reported heartbeats/actual heartbeats) × 100. Each case completed six trials at 35 s × 2, 25 s × 2, and 45 s × 2, with the order randomized between subjects.

#### The Time Estimation Task

The time estimation task was developed by Dunn et al. (2010) to overcome the influence of time estimation on interoceptive accuracy. In the task, participants are asked to count the number of seconds during a given period, and then the reported length was compared with the actual duration. Time estimation error rates were calculated in a manner similar to that of the heartbeat perception error rate and checked whether the error rates for both task were dissociated. Each case completed six trials at 23 s × 2, 40 s × 2, and 56 s × 2, with the order randomized between subjects.

#### Emotional Sensitivity Task

To estimate the emotional sensitivity of the cases, we prepared an experimental task that was identical to the task used in Terasawa et al. (2014). A detailed description of the task can be found in Terasawa et al. (2014). In this task, the cases were presented with a facial expression photo and were asked whether or not they felt emotion from the photos, and were asked to report the name of the emotion if they did feel emotion.

**FIGURE 2:**
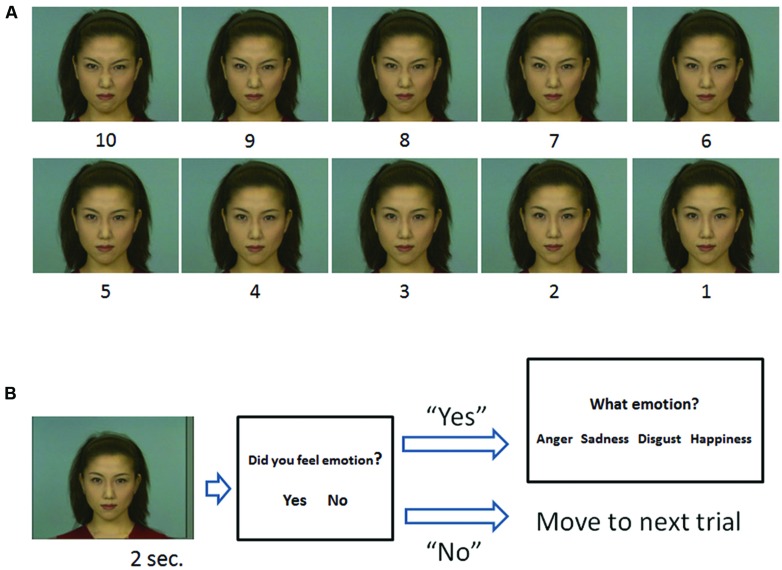
**The stimuli used in the task (A) and outline of a trial (B). (A)** Morphed continua photos ranging between neutral (stimulus 1) and 100% of each facial expression (stimulus 10), i.e., angry. **(B)** A stimulus was followed by a judgment about whether the stimulus elicited an emotion. If participants responded “yes,” options were presented and they were asked to choose the most appropriate emotion category.

We selected five photos each of a male individual and a female individual, with the following facial expressions: angry, sad, disgust, happiness, and neutral, from the Advanced Telecommunications Research Institute International Facial Expression Database (DB99). In addition to the original photos, morphed photos were prepared for the task. These photos were made in nine variations, with each variation having different percentages of the neutral and emotion expressions (angry, sad, disgust, and happiness), ranging from 10% neutral to 100% of each emotion. For example, Neutral (N) 90% – Happiness (H):10%, N:80% – H:20%, N:70% – H:30%, N:60% – H:40%, N:50% – H:50%, N:40% – H:60%, N:30% – H:70%, N:20% – H:80%, N:10% – H:90%, and N:0% – H:100% (**Figure 2A**). We did not use the original neutral picture (N:100% – H:0%) in this task, as every subject answered that they did not feel emotion from the picture.

In each trial, (1) a stimulus was presented for 2 s. (2) Participants were asked whether the stimulus made them feel an emotion or not. (3) If participants responded “Yes,” then four options were presented on the screen: anger, sadness, disgust, and happiness, and they were asked to choose the appropriate one for the emotion expressed by the stimulus. Because it was important to determine if the participants felt emotion, we asked them to respond “Yes” if they actually felt any emotion from the stimulus. Participants were clearly instructed to categorize emotion that the model expressed, not the one that they were experiencing. If they responded “No,” the options did not appear and the task moved on to the next trial after the presentation of a fixation point for 5 s (**Figure 2B**).

Each stimulus was presented five times in random order, thus there were 200 trials in total (4 emotion × 10 steps × 5 times). Original stimuli that fully expressed a certain emotion (e.g., 100% anger) were labeled as having an “emotion value of 10,” and neutral stimuli (e.g., 100% neutral) were labeled as having an “emotion value of 1.” Each step between emotion values 10 and 1, e.g., from 9 to 2, was labeled depending on the percentage of the emotional value present in the photo (**Figure 2A**).

The number of times that participants reported feeling emotions as a result of viewing each stimulus were calculated. We classified those stimuli that made participants feel an appropriate emotion at least three times out of five (i.e., at least 60% of the trials) as having sufficient emotional value to produce the emotional response.

The threshold of emotional value was posited to be located near the midpoint between stimuli that produced an emotional response less than three times and stimuli that produced an emotional response three times and more. When participants reported that they felt a certain emotion three times when viewing a stimulus with an emotion value of six and two times when viewing the stimulus with an emotion value of 5, we considered their threshold for emotional response to be 5.5.

## Results

### Emotional Sensitivity Task

**FIGURE 3:**
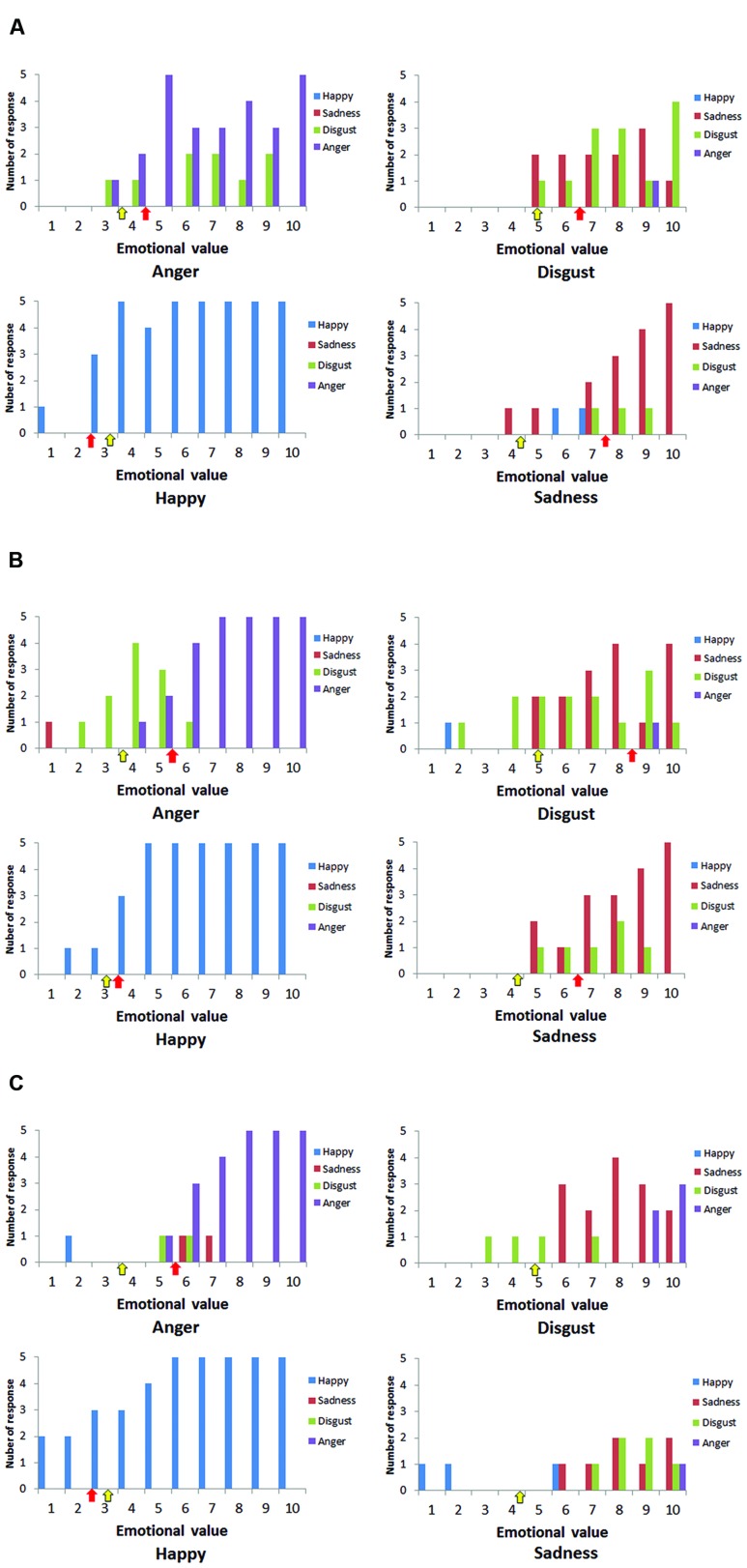
**Numbers of responses for happiness (blue), sadness (red), disgust (green), and anger (purple) for each stimulus. (A)** Case A, **(B)** Case B, and **(C)** Case C. Red arrows show the place where the thresholds of cases appeared, and yellow arrows show the averaged thresholds of healthy participants.

Case A’s responses toward each stimuli and thresholds of emotional value are shown in **Figure 3A**. The red arrows show the place where the thresholds appear. Yellow arrows show averaged thresholds of the age matched control group. The thresholds are also shown in **Table 1**. The responses of Cases B and C are shown in **Figures 3B,C**, respectively. As the three figures and the table show, all cases needed a higher emotional value to recognize anger than healthy participants. The same was true for sadness and disgust. Case C made too many error responses, such as false identification of disgust as sadness or sadness as neutral, which prevented the definition of thresholds for disgust and sadness.

**Table 1:** Thresholds of emotional value of the cases and healthy participants.

		Threshold	*t*-value	*p*	Zcc	Control
Case A	Anger	4.5	0.68	0.26	0.70	3.64 (1.23)
	Disgust	6.5	1.00	0.17	1.03	4.94 (1.51)
	Happiness	2.5	-0.55	0.30	-0.57	3.18 (1.20)
	Sadness	7.5^∗^	1.99	0.03	2.06	4.43 (1.49)
Case B	Anger	5.5+	1.46	0.08	1.51	
	Disgust	8.5^∗^	2.28	0.02	2.36	
	Happiness	3.5	0.26	0.40	0.27	
	Sadness	6.5	1.34	0.10	1.39	
Case C	Anger	5.5+	1.46	0.08	1.51	
	Disgust	-	-	-	-	
	Happiness	2.5	-0.55	0.30	-0.57	
	Sadness	-	-	-	-	

*SD in healthy participants are shown in parentheses. Zcc, effect size for simple t-test. ^∗^p < 0.05, ^+^p < 0.10.*

Although the numbers of case and control participants was small, we compared the thresholds of cases and the control group statistically. Crawford and Garthwaite (2002) and Crawford et al. (2010) reported statistical methods to compare scores of a single case and control group, and provided a program called Singlims_ES, which can be downloaded from http://homepages.abdn.ac.uk/j.crawford/pages/dept/Single_Case_Effect_Sizes.htm. This allowed us to test whether an individuals’ score was significantly different from a control sample. As a result, the threshold for anger in Cases B and C showed a trend for being higher than in the control group (*t* = 1.46, *p* < 0.10; **Table 1**). Case A showed a significantly higher threshold for sadness (*t* = 1.99, *p* < 0.05), while Case B showed a higher threshold for disgust (*t* = 2.28, *p* < 0.05). Not all thresholds of the cases were statistically higher than those of the control group, although the overall trends were the same, except for happiness.

The results support the idea that that insula lesions do not lead to impaired recognition of a specific category of emotion such as disgust, but rather that sensitivity to emotional value is blunted. However, the recognition of happy emotions was not affected by insula lesions because the cases showed similar sensitivity to happy faces as healthy participants.

### The Heartbeat Perception Task

Cases A and C completed the heartbeat perception task and the time estimation task, but B did not participate in the tasks. The error rates on the heartbeat perception task were 0.67 (Case A) and 0.99 (Case C), thus both cases showed very high error rates on this task. However, remarkably low error rates were observed on the time estimation task: 0.01 for Case A and 0.06 for Case C. High error rates on the heartbeat perception task indicate that lower interoceptive accuracy underlies the blunted sensitivity to others’ emotion measured by the emotional sensitivity task.

The control group also completed the heartbeat perception task, with a mean error rate of 0.70 ± 0.23. There were no differences between Case A compared with control group, while Case C showed significantly lower performance.

## Discussion

In this study, we examined the effects on emotional experience following brain lesions including the right anterior/middle insula cortex. Findings from the emotional sensitivity task and the heartbeat perception task indicated that the cases had blunted sensitivity to emotional expression and lower interoceptive accuracy. Some previous studies have suggested that insular lesions lead to selective impairment of disgust recognition (Calder et al., 2000; Adolphs et al., 2003; Borg et al., 2013); however, other studies have suggested that such lesions yield lower performance for discrimination between negative emotions (Adolphs et al., 2000; Dal Monte et al., 2013). Furthermore, other studies have proposed reduced arousal to emotional stimuli regardless of emotional valence (Berntson et al., 2011).

At the very least, our findings do not support the hypothesis positing that insula lesions cause the selective impairment of disgust recognition. Though Cases A and B needed higher emotional value to recognize disgust compared with healthy participants, they could properly recognize disgust when the stimuli had higher values. Case C could not recognize disgust, but also sadness, thus the impairment cannot be considered selective. However, all cases showed some degree of difficulty in recognizing emotions from stimuli.

Unique patterns of error response can be seen when focused on **Figures 3A–C**; these are (i) misidentification of anger as disgust or sadness and (ii) misidentification of disgust as sadness. Based on a circumplex theory of emotion and insular function for recognizing arousal level, these patterns are very compelling. According to the circumplex theory of emotion (Russell, 1980), emotions can be plotted on a plane defined by two dimensions: arousal and valence. Anger, disgust, and sadness have similar emotional valence, but differences in arousal level discriminate these three emotions. Attenuation of recognized arousal level may lead to the error patterns that can be seen in the present study, because the arousal level of anger is the highest, disgust is middle, and sadness is the lowest. As the insular cortex is related to the recognition of arousal level, the error patterns may suggest that the cases could understand the emotional valence of stimuli, but that they had difficulty in sensing arousal level from the stimuli. Thus, they identified emotions (e.g., anger) that have high arousal level as less aroused emotions (e.g., sadness). Arousal level is closely connected to bodily responses such as cardiovascular activity, respiration, and body temperature. If interoceptive awareness for these responses underlies recognition of arousal level, it is reasonable to assume that insular lesions lead to reduced arousal level, and then to the altered emotional recognition performance observed in the present study.

Few previous studies have examined whether insular lesions lead to a decline in interoceptive accuracy. Khalsa et al. (2009) revealed that heartbeat sensation was not significantly affected by bilateral insular lesion, until an anesthetic was otherwise applied to the skin. However, Ronchi et al. (2015) reported that resection of selective right neoplastic insular lesions led to an increment of error rates on the heartbeat perception task, a result identical to that of our study. In particular, the resection boosted the error rates from 0.18 to 0.39. The patient of Ronchi et al. (2015) had higher interoceptive accuracy before resection, but that was not the case after resection. We did not measure interoceptive accuracy of cases prior to the lesion in the present study, and the heartbeat perception task showed rather large individual differences. In fact, although Case C showed remarkably low performance in the heartbeat perception task, the performance of Case A was identical to the control group. Thus, the high error rates of Case C could not be easily attributed to the right anterior insular lesion. As suggested by Damasio et al. (2012) and Couto et al. (2013), we considered the function of the insular subcortical network for the performance. The lesion area of Case A was rather selective, suggesting that the subcortical network should be largely preserved. The effect of coexisting somatosensory routes of interoception should also be considered (Khalsa et al., 2009; Couto et al., 2014). Findings from a series of previous studies and our results remain insufficient to fully elucidate the role of the insula for interoception and emotional awareness. Further testing of insular lesion cases is required to understand whether insular lesions lead to a decline in interoceptive accuracy and emotional awareness.

The reported episodes of the cases’ daily life are also interesting. None of the three cases had a major problem with their memory and intelligence, but all reported episodes that indicate altered emotional experience. An absence of feelings of anxiety, observed in Cases B and C, is remarkable, because anxiety is considered to be closely related to interoception and the insular cortex is one of the neural correlates for this relationship (Paulus and Stein, 2006; Simmons et al., 2011; Terasawa et al., 2013a). These studies propose that amplified sensitivity to bodily information enables us to detect subtle internal bodily changes and evokes anxiety, because anxiety states are closely related to sympathetic activities. If the exaggerated attention to interoceptive information is assumed to yield anxious feelings, then conversely the unavailability of interoceptive information may reduce anxiety.

However, there were no significant differences between the cases and healthy participants for the thresholds of emotional value for happiness. If attenuated interoceptive accuracy lowers arousal level, this trend should also be observed for happiness recognition. We posit three possible reasons for this finding. The first hypothesis is that the right insula specifically underlies negative emotion processing, not that for positive emotion. Craig (2005) suggested this asymmetrical function of the insula, and proposed associations of the right anterior insula with energy expenditure (arousal) feelings, such as risk and depression, and the left insula with energy enrichment (affiliation) feelings, such as social bonding. His suggestion may support the notion that positive emotion is related to left insular activity rather than the right insula. However, some studies have reported that highly aroused emotions recruit anterior insula activity regardless of valence (Iaria et al., 2008; Moriguchi et al., 2014). These studies imply that the laterality of insular function is still controversial, and this idea seems insufficient for explaining our findings about happiness. The second hypothesis is that people have a tendency to process happy faces preferentially more than other emotions. Calvo and Nummenmaa (2008) observed that happy faces need less time to be detected precisely compared with fearful, angry, sad, surprised, and disgusted faces. The findings suggest that sensitivity to happy faces predominates over the blunting of emotion recognition based on the insula lesion, thus recognition of happy emotions was maintained. The third hypothesis concerns a technical issue, that there is only one option for positive valence emotion. If cases could understand valence properly, there is no possibility to misidentify happy as other emotions, but the possibilities were high for negative emotions, because there are three options for negative emotions. Our results are not detailed enough to decide which is the most plausible hypothesis, but the findings suggest that the cases could understand emotional valence properly and that the effect of interoception would be rather small on recognition of the valence.

All three cases showed lower performance in the WCST, and the number of perseveration error responses was quite high in particular. Neuroimaging studies designate the involvement of the insula cortex in various cognitive functions such as attention or memory, thus it is possible to posit that insula lesions affected performance through basic cognitive functions (Kurth et al., 2010). However, we need to note that the cases’ lesions extended to the right DLPFC, which is well known for neural correlates for executing the WCST (Buchsbaum et al., 2005; Lie et al., 2006). The effects of the cases’ DLPFC lesions would be to induce lower performance on the WCST. Since DLPFC is known as an important region for appraisal or regulation processes of emotion, we should consider possibility that the DLPFC lesion may affect the performance of the emotional sensitivity task. Recent neuropsychological study (Jenkins et al., 2014) which used morphed expression photos showed the effect of DLPFC lesion on recognition of emotional expression was restricted. Though the effect of DLPFC lesion on the emotional sensitivity task would be limited, further careful investigations should be conducted to clarify the possible sole effect of DLPFC lesion.

Thus, extended lesion areas in the cases prevent us from concluding that the findings originated from the insular lesion selectively, which is a limitation of the present study. Future studies with more selective insula lesion cases are required. Recent reports suggest that the insular cortex can be divided into anterior/middle/posterior and ventral/dorsal regions, with differing cognitive functions in these regions. For example, Kurth et al. (2010) suggested that the ventral region was important for social-emotional function, the anterior dorsal region for cognitive function, and the medial to posterior regions for sensorimotor function. However, it was also reported that interoceptive processing is reliant on the posterior region, while exteroceptive processing relies on the anterior region (Farb et al., 2013). These inconsistent findings suggest that further neuropsychological studies focusing on the lesion area in the insula and its impact on cognitive function are required. Unfortunately, the lesion areas of the three cases in the present study were not restricted to a particular region.

Despite the small number of cases, our findings suggest that perceiving interoceptive information may modulate the contents of emotional experience, and the notion is consistent with some previous findings. In the future, studies with more lesion cases will be beneficial for understanding the causality between interoception and subjective emotion through neural substrates.

## Conflict of Interest Statement

The authors declare that the research was conducted in the absence of any commercial or financial relationships that could be construed as a potential conflict of interest.
